# Supplementary material for: Genetic diversity in two Plasmodium vivax protein ligands for reticulocyte invasion
Source: PLoS Negl Trop Dis. 2018 Oct 22;12(10):e0006555. doi: 10.1371/journal.pntd.0006555 (PMC6211765; doi:10.1371/journal.pntd.0006555)
Supplement: S1 Table — PVX_110810, Pv_Sal1_chr06:976,329–980,090 (+) was used as reference. (DOCX) [file pntd.0006555.s001.docx]

Mutations specific to Cambodia, to Madagascar or observed in both countries are presented in cells colored in red, green and blue, respectively.
