# Supplementary material for: Genetic diversity in two Plasmodium vivax protein ligands for reticulocyte invasion
Source: PLoS Negl Trop Dis. 2018 Oct 22;12(10):e0006555. doi: 10.1371/journal.pntd.0006555 (PMC6211765; doi:10.1371/journal.pntd.0006555)
Supplement: S2 Table — P. vivax isolate C127 nEBP gene, KC987954.1 was used as reference. (DOCX) [file pntd.0006555.s002.docx]

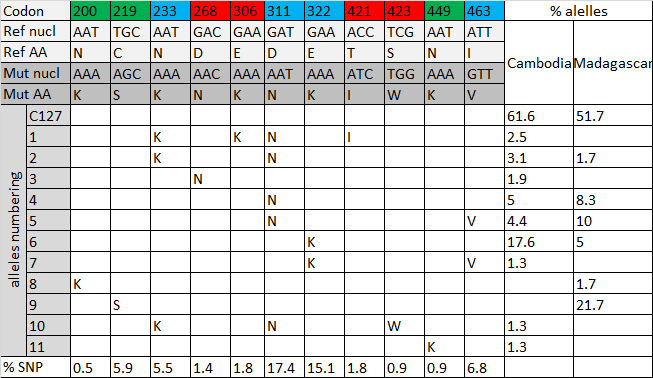


Mutations specific to Cambodia, to Madagascar or observed in both countries are presented in cells colored in red, green and blue, respectively.
