# Supplementary material for: Genetic diversity in two Plasmodium vivax protein ligands for reticulocyte invasion
Source: PLoS Negl Trop Dis. 2018 Oct 22;12(10):e0006555. doi: 10.1371/journal.pntd.0006555 (PMC6211765; doi:10.1371/journal.pntd.0006555)
Supplement: S3 Table — (DOCX) [file pntd.0006555.s003.docx]

| **PCR** | **Primers name** | **Sequence** |
| --- | --- | --- |
| PvDBP CNV | CN_PvDBP_F | 5′-AATTATAAGAGAAAACGTCGGGAAAG-3′ |
|  | CN_PvDBP_R | 5′-ACCAAATTCGTAAGTTCCTTCATACA -3′ |
| PvEBP CNV | CN_PvEBP_F | 5’-GGGGGAAGTACGAAAGATGAA-3’ |
|  | CN_PvEBP_R | 5’-CATCCCTTTGACCATTGACC-3’ |
| Housekeeping gene CNV | CN_β-tubulin_F | 5′-CATGTTCGTTAAGATTTCCTGGT-3′ |
|  | CN_β-tubulin_R | 5′-GTTAGTGGTGCAAAACCAATCA-3′ |
| Synthetic genes for CNV (standard curve) | PvDBP | GAAAAACTGTAATTATAAGAGAAAACGTCGGGAAAGAGATTGGGACTGTAACACTAAGAAGGATGTTTGTATACCAGATCGAAGATATCAATTATGTATGAAGGAACTTACGAATTTGGTAAATAATACA |
|  | PvEBP | GGTGTAAAAATTGCGGGGGGAAGTACGAAAGATGAAAATCGGGGAGATCCAACATCGCACGAAATAAGATCGCATGAAGGAGGAAGTAGAGCAGCGGTCAATGGTCAAAGGGATGACGCTGGACGTGTTC |
|  | β-TubulinPv | CAGGAGTTACATGTTCGTTAAGATTTCCTGGTCAGTTAAATTCTGATTTGAGAAAATTAGCTGTCAATTTAATTCCCTTCCCAAGACTCCACTTTTTTATGATTGGTTTTGCACCACTAACAAGCAGAGG |
| PvDBP SNP | PvDBPsd_PF : | 5'-GCATGAGGGAAATTCTCGTA-3' |
|  | PvDBPsd_PR: | 5'-CGTTAAATTCATCTAACTCCTGTTT-3' |
|  | PvDBPsd_NF: | 5'-GAATGGTGGCAATCCTTACG-3' |
|  | PvDBPsd_NR: | 5'-TCTGAACCTTTTCTGCGTTTT-3' |
| PvEBP SNP | PvEBPsd_PF: | 5'-CCAACGAAAGGAGATGAGAA-3' |
|  | PvEBPsd_PR: | 5'-TCCACTTCGGTGGAATCTAT-3' |
|  | PvEBPsd_NF | 5'-ATGCAGAGGGTGCAGATACC-3' |
|  | PvEBPsd_NR | 5'-TTCCCATTCTCATCGACCTC-3' |
| Duffy genotyping | PCR_Fy_F (PCR Fy) | 5'-GTGGGGTAAGGCTTCCTGAT-3' |
|  | PCR_Fy_R (PCR Fy) | 5'-CAGAGCTGCGAGTGCTACCT-3' |
|  | PCR_Fy_F (PCR Nested GATA) | 5'-GTGGGGTAAGGCTTCCTGAT-3' |
|  | Nested_GATA (PCR Nested GATA) | 5'-CAAACAGCAGGGGAAATGAG-3' |
|  | Nested_Fy_SNP (PCR Nested Fy SNP) | 5'-CTTCCGGTGTAACTCTGATGG-3' |
|  | PCR_Fy_R (PCR Nested Fy SNP) | 5'-CAGAGCTGCGAGTGCTACCT-3' |

# *PvDBP* and *PvEBP* copy number determination, protocols. *PvDBP* (PVP01_0623800) and *PvEBP* (PVP01_0102300) copy numbers were measured by qPCR using a CFX96 real-time PCR machine (Bio-Rad). As a reference, we used the single copy β*-tubulin* (PVX_094635) gene.

# *PvDBP copy number.*

# Quantitative PCR (qPCR) was carried out in 20 μl volumes in a 96-well plate containing 1X HOT FIREPol EvaGreen qPCR Mix Plus (Solis BioDyne, Estonia), 0.5 μM of each forward and reverse primer and 2 μl of template DNA. Amplifications were performed under the following conditions: 95°C for 15min, followed by 45 cycles of 95°C for 15s, 60°C for 20s, and 72°C for 20s.

# *PvDBP* copy number of each sample was measured in triplicate relative to a standard curve using six standards of mixed synthetic gene fragments (Eurofins Genomics, Ebersberg, Germany) (see Table S3). The six standards of mixed synthetic gene fragments were: standard 1 (1:1 molar ratio of *PvDBP &* β-tubulin), standard 2 (2:1 molar ratio of *PvDBP &* β-tubulin), standard 3 (3:1 molar ratio of *PvDBP &* β-tubulin), standard 4 (4:1 molar ratio of *PvDBP &* β-tubulin) and standard 5 (5:1 molar ratio of *PvDBP &* β-tubulin), standard 6 (6:1 molar ratio of *PvDBP &* β-tubulin). A *P. vivax* Cambodian isolate (one copy of *PvDBP*) was included in each run as control. *PvDBP* copy number was calculated by the 2-ΔCt method (ΔCt = Ct *PvDBP* - Ct *Pvβ-tubulin* where Ct is the threshold cycle) and deduced from the standard curve. A *PvDBP* copy number >1.5 was defined as an amplification of the gene. Amplification efficiencies of the *PvDBP* and the *Pvβ-tubulin* genes, measured using ten-fold dilutions of a *P. vivax* Cambodian isolate, were similar (>90%).

# *PvEBP copy number.*

# qPCR was carried out in 20 μl volumes in a 96-well plate containing 1X HOT FIREPol EvaGreen qPCR Mix Plus (Solis BioDyne, Estonia), 0.5 μM of each forward and reverse primer and 2 μl of template DNA. Amplifications were performed under the following conditions: 95°C for 15min, followed by 45 cycles of 95°C for 15s, 60°C for 20s, and 72°C for 20s.

# *PvEBP* copy number of each sample was measured in triplicate relative to a standard curve using six standards of mixed synthetic gene fragments (Eurofins Genomics, Ebersberg, Germany) (see Table S3). The six standards of mixed synthetic gene fragments were: standard 1 (1:1 molar ratio of *PvEBP &* β-tubulin), standard 2 (2:1 molar ratio of *PvEBP &* β-tubulin), standard 3 (3:1 molar ratio of *PvEBP &* β-tubulin), standard 4 (4:1 molar ratio of *PvEBP &* β-tubulin) and standard 5 (5:1 molar ratio of *PvEBP &* β-tubulin), standard 6 (6:1 molar ratio of *PvBP &* β-tubulin). A *P. vivax* Cambodian isolate (one copy of *PvEBP*) was included in each run as control. *PvEBP* copy number was calculated by the 2-ΔCt method (ΔCt = Ct *PvEBP* - Ct *Pvβ-tubulin* where Ct is the threshold cycle) and deduced from the standard curve. A *PvEBP* copy number >1.5 was defined as an amplification of the gene. Amplification efficiencies of the *PvEBP* and the *Pvβ-tubulin* genes, measured using ten-fold dilutions of a *P. vivax* Cambodian isolate, were similar (>90%).
