# Supplementary material for: Genetic diversity in two Plasmodium vivax protein ligands for reticulocyte invasion
Source: PLoS Negl Trop Dis. 2018 Oct 22;12(10):e0006555. doi: 10.1371/journal.pntd.0006555 (PMC6211765; doi:10.1371/journal.pntd.0006555)
Supplement: S4 Table — (DOCX) [file pntd.0006555.s004.docx]

| **Country** | **ID** | **Year of collection** | **Location (province)** | **Duffy genotyping (GATA box)** | **PvDBP unrounded gene copy number** | **PvDBP allele number** | **PvEBP unrounded gene copy number** | **PvEBP allele number** |
| --- | --- | --- | --- | --- | --- | --- | --- | --- |
| Cambodia | KH001 | 2003 | Battambang | N/A | 0.71 | N/A | N/A | N/A |
| Cambodia | KH002 | 2003 | Battambang | N/A | 1.67 | N/A | N/A | N/A |
| Cambodia | KH003 | 2004 | Ratanakiri | N/A | 0.56 | N/A | N/A | N/A |
| Cambodia | KH004 | 2004 | Ratanakiri | N/A | 0.57 | N/A | N/A | N/A |
| Cambodia | KH005 | 2004 | Ratanakiri | N/A | 0.63 | N/A | N/A | N/A |
| Cambodia | KH006 | 2004 | Battambang | N/A | 0.76 | N/A | N/A | N/A |
| Cambodia | KH007 | 2004 | Ratanakiri | N/A | 0.85 | N/A | N/A | N/A |
| Cambodia | KH008 | 2004 | Ratanakiri | N/A | 0.88 | N/A | N/A | N/A |
| Cambodia | KH009 | 2004 | Ratanakiri | N/A | 0.95 | N/A | N/A | N/A |
| Cambodia | KH010 | 2004 | Ratanakiri | N/A | 0.96 | N/A | N/A | N/A |
| Cambodia | KH011 | 2004 | Battambang | N/A | 0.99 | N/A | N/A | N/A |
| Cambodia | KH012 | 2004 | Ratanakiri | N/A | 1.03 | N/A | N/A | N/A |
| Cambodia | KH013 | 2004 | Battambang | N/A | 1.03 | N/A | N/A | N/A |
| Cambodia | KH014 | 2004 | Battambang | N/A | 1.05 | N/A | N/A | N/A |
| Cambodia | KH015 | 2004 | Ratanakiri | N/A | 1.06 | N/A | N/A | N/A |
| Cambodia | KH016 | 2004 | Battambang | N/A | 1.09 | N/A | N/A | N/A |
| Cambodia | KH017 | 2004 | Battambang | N/A | 1.12 | N/A | N/A | N/A |
| Cambodia | KH018 | 2004 | Battambang | N/A | 1.12 | N/A | N/A | N/A |
| Cambodia | KH019 | 2004 | Battambang | N/A | 1.17 | N/A | N/A | N/A |
| Cambodia | KH020 | 2004 | Battambang | N/A | 1.19 | N/A | N/A | N/A |
| Cambodia | KH021 | 2004 | Battambang | N/A | 1.20 | N/A | N/A | N/A |
| Cambodia | KH022 | 2004 | Battambang | N/A | 1.22 | N/A | N/A | N/A |
| Cambodia | KH023 | 2004 | Battambang | N/A | 1.22 | N/A | N/A | N/A |
| Cambodia | KH024 | 2004 | Battambang | N/A | 1.25 | N/A | N/A | N/A |
| Cambodia | KH025 | 2004 | Battambang | N/A | 1.27 | N/A | N/A | N/A |
| Cambodia | KH026 | 2004 | Battambang | N/A | 1.27 | N/A | N/A | N/A |
| Cambodia | KH027 | 2004 | Battambang | N/A | 1.28 | N/A | N/A | N/A |
| Cambodia | KH028 | 2004 | Battambang | N/A | 1.30 | N/A | N/A | N/A |
| Cambodia | KH029 | 2004 | Battambang | N/A | 1.31 | N/A | N/A | N/A |
| Cambodia | KH030 | 2004 | Battambang | N/A | 1.34 | N/A | N/A | N/A |
| Cambodia | KH031 | 2004 | Battambang | N/A | 1.34 | N/A | N/A | N/A |
| Cambodia | KH032 | 2004 | Battambang | N/A | 1.36 | N/A | N/A | N/A |
| Cambodia | KH033 | 2004 | Battambang | N/A | 1.36 | N/A | N/A | N/A |
| Cambodia | KH034 | 2004 | Battambang | N/A | 1.37 | N/A | N/A | N/A |
| Cambodia | KH035 | 2004 | Battambang | N/A | 1.38 | N/A | N/A | N/A |
| Cambodia | KH036 | 2004 | Battambang | N/A | 1.38 | N/A | N/A | N/A |
| Cambodia | KH037 | 2004 | Battambang | N/A | 1.38 | N/A | N/A | N/A |
| Cambodia | KH038 | 2004 | Ratanakiri | N/A | 1.38 | N/A | N/A | N/A |
| Cambodia | KH039 | 2004 | Ratanakiri | N/A | 1.39 | N/A | N/A | N/A |
| Cambodia | KH040 | 2004 | Battambang | N/A | 1.41 | N/A | N/A | N/A |
| Cambodia | KH041 | 2004 | Ratanakiri | N/A | 1.43 | N/A | N/A | N/A |
| Cambodia | KH042 | 2004 | Ratanakiri | N/A | 1.51 | N/A | N/A | N/A |
| Cambodia | KH043 | 2004 | Battambang | N/A | 1.53 | N/A | N/A | N/A |
| Cambodia | KH044 | 2004 | Battambang | N/A | 1.62 | N/A | N/A | N/A |
| Cambodia | KH045 | 2004 | Ratanakiri | N/A | 1.73 | N/A | N/A | N/A |
| Cambodia | KH046 | 2004 | Battambang | N/A | 3.03 | N/A | N/A | N/A |
| Cambodia | KH047 | 2004 | Battambang | N/A | 3.20 | N/A | N/A | N/A |
| Cambodia | KH048 | 2004 | Ratanakiri | N/A | 4.11 | N/A | N/A | N/A |
| Cambodia | KH049 | 2011 | Battambang | N/A | 0.65 | N/A | N/A | N/A |
| Cambodia | KH050 | 2011 | Mondulkiri | N/A | 0.67 | N/A | N/A | N/A |
| Cambodia | KH051 | 2011 | Pailin | N/A | 0.79 | N/A | N/A | N/A |
| Cambodia | KH052 | 2011 | Mondulkiri | N/A | 0.84 | N/A | N/A | N/A |
| Cambodia | KH053 | 2011 | Pursat | N/A | 0.87 | N/A | N/A | N/A |
| Cambodia | KH054 | 2011 | Mondulkiri | N/A | 0.91 | N/A | N/A | N/A |
| Cambodia | KH055 | 2011 | Mondulkiri | N/A | 0.92 | N/A | N/A | N/A |
| Cambodia | KH056 | 2011 | Battambang | N/A | 0.93 | N/A | N/A | N/A |
| Cambodia | KH057 | 2011 | Mondulkiri | N/A | 0.98 | N/A | N/A | N/A |
| Cambodia | KH058 | 2011 | Ratanakiri | N/A | 1.14 | N/A | N/A | N/A |
| Cambodia | KH059 | 2011 | Ratanakiri | N/A | 1.16 | N/A | N/A | N/A |
| Cambodia | KH060 | 2011 | Pailin | N/A | 1.17 | N/A | N/A | N/A |
| Cambodia | KH061 | 2011 | Battambang | N/A | 1.18 | N/A | N/A | N/A |
| Cambodia | KH062 | 2011 | Ratanakiri | N/A | 1.23 | N/A | N/A | N/A |
| Cambodia | KH063 | 2011 | Ratanakiri | N/A | 1.24 | N/A | N/A | N/A |
| Cambodia | KH064 | 2011 | Ratanakiri | N/A | 1.25 | N/A | N/A | N/A |
| Cambodia | KH065 | 2011 | Battambang | N/A | 1.27 | N/A | N/A | N/A |
| Cambodia | KH066 | 2011 | Battambang | N/A | 1.27 | N/A | N/A | N/A |
| Cambodia | KH067 | 2011 | Battambang | N/A | 1.30 | N/A | N/A | N/A |
| Cambodia | KH068 | 2011 | Battambang | N/A | 1.32 | N/A | N/A | N/A |
| Cambodia | KH069 | 2011 | Pursat | N/A | 1.33 | N/A | N/A | N/A |
| Cambodia | KH070 | 2011 | Battambang | N/A | 1.36 | N/A | N/A | N/A |
| Cambodia | KH071 | 2011 | Ratanakiri | N/A | 1.38 | N/A | N/A | N/A |
| Cambodia | KH072 | 2011 | Battambang | N/A | 1.39 | N/A | N/A | N/A |
| Cambodia | KH073 | 2011 | Ratanakiri | N/A | 1.42 | N/A | N/A | N/A |
| Cambodia | KH074 | 2011 | Mondulkiri | N/A | 1.45 | N/A | N/A | N/A |
| Cambodia | KH075 | 2011 | Ratanakiri | N/A | 1.48 | N/A | N/A | N/A |
| Cambodia | KH076 | 2011 | Ratanakiri | N/A | 1.54 | N/A | N/A | N/A |
| Cambodia | KH077 | 2011 | Battambang | N/A | 1.57 | N/A | N/A | N/A |
| Cambodia | KH078 | 2011 | Pailin | N/A | 1.59 | N/A | N/A | N/A |
| Cambodia | KH079 | 2011 | Battambang | N/A | 1.65 | N/A | N/A | N/A |
| Cambodia | KH080 | 2011 | Pursat | N/A | 1.65 | N/A | N/A | N/A |
| Cambodia | KH081 | 2011 | Battambang | N/A | 1.71 | N/A | N/A | N/A |
| Cambodia | KH082 | 2011 | Battambang | N/A | 1.79 | N/A | N/A | N/A |
| Cambodia | KH083 | 2011 | Battambang | N/A | 1.88 | N/A | N/A | N/A |
| Cambodia | KH084 | 2011 | Pailin | N/A | 1.97 | N/A | N/A | N/A |
| Cambodia | KH085 | 2011 | Battambang | N/A | 2.00 | N/A | N/A | N/A |
| Cambodia | KH086 | 2011 | Pursat | N/A | 2.02 | N/A | N/A | N/A |
| Cambodia | KH087 | 2011 | Ratanakiri | N/A | 2.03 | N/A | N/A | N/A |
| Cambodia | KH088 | 2011 | Ratanakiri | N/A | 2.07 | N/A | N/A | N/A |
| Cambodia | KH089 | 2011 | Pursat | N/A | 2.08 | N/A | N/A | N/A |
| Cambodia | KH090 | 2011 | Pailin | N/A | 2.10 | N/A | N/A | N/A |
| Cambodia | KH091 | 2011 | Battambang | N/A | 2.27 | N/A | N/A | N/A |
| Cambodia | KH092 | 2011 | Ratanakiri | N/A | 2.60 | N/A | N/A | N/A |
| Cambodia | KH093 | 2011 | Ratanakiri | N/A | 2.61 | N/A | N/A | N/A |
| Cambodia | KH094 | 2011 | Mondulkiri | N/A | 2.68 | N/A | N/A | N/A |
| Cambodia | KH095 | 2011 | Ratanakiri | N/A | 2.89 | N/A | N/A | N/A |
| Cambodia | KH096 | 2011 | Ratanakiri | N/A | 2.99 | N/A | N/A | N/A |
| Cambodia | KH097 | 2011 | Ratanakiri | N/A | 3.04 | N/A | N/A | N/A |
| Cambodia | KH098 | 2011 | Ratanakiri | N/A | 3.07 | N/A | N/A | N/A |
| Cambodia | KH099 | 2011 | Mondulkiri | N/A | 3.16 | N/A | N/A | N/A |
| Cambodia | KH100 | 2012 | Ratanakiri | N/A | 0.57 | N/A | N/A | N/A |
| Cambodia | KH101 | 2012 | Mondulkiri | N/A | 0.60 | N/A | N/A | N/A |
| Cambodia | KH102 | 2012 | Battambang | N/A | 0.66 | N/A | N/A | N/A |
| Cambodia | KH103 | 2012 | Pailin | N/A | 0.67 | N/A | N/A | N/A |
| Cambodia | KH104 | 2012 | Ratanakiri | N/A | 0.68 | N/A | N/A | N/A |
| Cambodia | KH105 | 2012 | Battambang | N/A | 0.85 | N/A | N/A | N/A |
| Cambodia | KH106 | 2012 | Battambang | N/A | 0.90 | N/A | N/A | N/A |
| Cambodia | KH107 | 2012 | Ratanakiri | N/A | 0.92 | N/A | N/A | N/A |
| Cambodia | KH108 | 2012 | Battambang | N/A | 0.93 | N/A | N/A | N/A |
| Cambodia | KH109 | 2012 | Ratanakiri | N/A | 0.95 | N/A | N/A | N/A |
| Cambodia | KH110 | 2012 | Pailin | N/A | 0.99 | N/A | N/A | N/A |
| Cambodia | KH111 | 2012 | Battambang | N/A | 1.02 | N/A | N/A | N/A |
| Cambodia | KH112 | 2012 | Pursat | N/A | 1.04 | N/A | N/A | N/A |
| Cambodia | KH113 | 2012 | Mondulkiri | N/A | 1.07 | N/A | N/A | N/A |
| Cambodia | KH114 | 2012 | Ratanakiri | N/A | 1.07 | N/A | N/A | N/A |
| Cambodia | KH115 | 2012 | Ratanakiri | N/A | 1.11 | N/A | N/A | N/A |
| Cambodia | KH116 | 2012 | Pursat | N/A | 1.12 | N/A | N/A | N/A |
| Cambodia | KH117 | 2012 | Ratanakiri | N/A | 1.15 | N/A | N/A | N/A |
| Cambodia | KH118 | 2012 | Ratanakiri | N/A | 1.15 | N/A | N/A | N/A |
| Cambodia | KH119 | 2012 | Battambang | N/A | 1.17 | N/A | N/A | N/A |
| Cambodia | KH120 | 2012 | Battambang | N/A | 1.19 | N/A | N/A | N/A |
| Cambodia | KH121 | 2012 | Battambang | N/A | 1.20 | N/A | N/A | N/A |
| Cambodia | KH122 | 2012 | Battambang | N/A | 1.20 | N/A | N/A | N/A |
| Cambodia | KH123 | 2012 | Ratanakiri | N/A | 1.21 | N/A | N/A | N/A |
| Cambodia | KH124 | 2012 | Battambang | N/A | 1.24 | N/A | N/A | N/A |
| Cambodia | KH125 | 2012 | Battambang | N/A | 1.26 | N/A | N/A | N/A |
| Cambodia | KH126 | 2012 | Battambang | N/A | 1.28 | N/A | N/A | N/A |
| Cambodia | KH127 | 2012 | Battambang | N/A | 1.3 | N/A | N/A | N/A |
| Cambodia | KH128 | 2012 | Ratanakiri | N/A | 1.31 | N/A | N/A | N/A |
| Cambodia | KH129 | 2012 | Pursat | N/A | 1.32 | N/A | N/A | N/A |
| Cambodia | KH130 | 2012 | Ratanakiri | N/A | 1.37 | N/A | N/A | N/A |
| Cambodia | KH131 | 2012 | Ratanakiri | N/A | 1.46 | N/A | N/A | N/A |
| Cambodia | KH132 | 2012 | Mondulkiri | N/A | 1.48 | N/A | N/A | N/A |
| Cambodia | KH133 | 2012 | Ratanakiri | N/A | 1.65 | N/A | N/A | N/A |
| Cambodia | KH134 | 2012 | Ratanakiri | N/A | 1.65 | N/A | N/A | N/A |
| Cambodia | KH135 | 2012 | Battambang | N/A | 1.77 | N/A | N/A | N/A |
| Cambodia | KH136 | 2012 | Ratanakiri | N/A | 2.13 | N/A | N/A | N/A |
| Cambodia | KH137 | 2012 | Ratanakiri | N/A | 2.24 | N/A | N/A | N/A |
| Cambodia | KH138 | 2012 | Battambang | N/A | 2.24 | N/A | N/A | N/A |
| Cambodia | KH139 | 2012 | Ratanakiri | N/A | 2.35 | N/A | N/A | N/A |
| Cambodia | KH140 | 2012 | Pursat | N/A | 2.40 | N/A | N/A | N/A |
| Cambodia | KH141 | 2012 | Battambang | N/A | 2.55 | N/A | N/A | N/A |
| Cambodia | KH142 | 2012 | Battambang | N/A | 2.88 | N/A | N/A | N/A |
| Cambodia | KH143 | 2012 | Pailin | N/A | 2.96 | N/A | N/A | N/A |
| Cambodia | KH144 | 2012 | Ratanakiri | N/A | 3.20 | N/A | N/A | N/A |
| Cambodia | KH145 | 2012 | Pursat | N/A | 3.22 | N/A | N/A | N/A |
| Cambodia | KH146 | 2012 | Pailin | N/A | 3.42 | N/A | N/A | N/A |
| Cambodia | KH147 | 2012 | Ratanakiri | N/A | 4.80 | N/A | N/A | N/A |
| Cambodia | KH148 | 2013 | Pailin | N/A | N/A | 14 | N/A | 6 |
| Cambodia | KH149 | 2013 | Pailin | N/A | N/A | N/A | N/A | 6 |
| Cambodia | KH150 | 2013 | Kampot | N/A | N/A | 12 | N/A | 7 |
| Cambodia | KH151 | 2013 | Kampot | N/A | N/A | 3 | N/A | C127 |
| Cambodia | KH152 | 2013 | Kampot | N/A | N/A | 6 | N/A | C127 |
| Cambodia | KH153 | 2013 | Kampot | N/A | N/A | 6 | N/A | C127 |
| Cambodia | KH154 | 2013 | Pailin | N/A | N/A | 6 | N/A | N/A |
| Cambodia | KH155 | 2013 | Pailin | N/A | N/A | 8 | N/A | N/A |
| Cambodia | KH156 | 2013 | Pailin | N/A | 0.80 | 2 | N/A | 1 |
| Cambodia | KH157 | 2013 | Kampot | N/A | N/A | 6 | 0.56 | 4 |
| Cambodia | KH158 | 2013 | Pailin | N/A | N/A | 10 | 0.57 | 4 |
| Cambodia | KH159 | 2013 | Kampot | N/A | N/A | 12 | 1.46 | 4 |
| Cambodia | KH160 | 2013 | Kampot | N/A | 1.02 | N/A | N/A | 4 |
| Cambodia | KH161 | 2013 | Kampot | N/A | 0.73 | 3 | N/A | 5 |
| Cambodia | KH162 | 2013 | Pailin | N/A | 1.29 | 3 | N/A | 6 |
| Cambodia | KH163 | 2013 | Pailin | N/A | 0.60 | 9 | N/A | 6 |
| Cambodia | KH164 | 2013 | Kampot | N/A | 0.87 | N/A | N/A | 6 |
| Cambodia | KH165 | 2013 | Pailin | N/A | N/A | 2 | 1.39 | C127 |
| Cambodia | KH166 | 2013 | Pailin | N/A | 1.49 | 3 | N/A | C127 |
| Cambodia | KH167 | 2013 | Kampot | N/A | N/A | 3 | 0.83 | C127 |
| Cambodia | KH168 | 2013 | Kampot | N/A | N/A | 3 | 0.75 | C127 |
| Cambodia | KH169 | 2013 | Pailin | N/A | N/A | 3 | 1.43 | C127 |
| Cambodia | KH170 | 2013 | Pailin | N/A | N/A | 4 | 0.97 | C127 |
| Cambodia | KH171 | 2013 | Kampot | N/A | N/A | 5 | 1.49 | C127 |
| Cambodia | KH172 | 2013 | Kampot | N/A | 0.90 | 7 | N/A | C127 |
| Cambodia | KH173 | 2013 | Pailin | N/A | N/A | 10 | 1.06 | C127 |
| Cambodia | KH174 | 2013 | Pailin | N/A | 0.55 | 11 | N/A | C127 |
| Cambodia | KH175 | 2013 | Kampot | N/A | N/A | 14 | 1.00 | C127 |
| Cambodia | KH176 | 2013 | Pailin | N/A | 0.61 | N/A | N/A | C127 |
| Cambodia | KH177 | 2013 | Kampot | N/A | 0.72 | N/A | N/A | C127 |
| Cambodia | KH178 | 2013 | Kampot | N/A | 1.05 | N/A | N/A | C127 |
| Cambodia | KH179 | 2013 | Kampot | N/A | 1.25 | N/A | N/A | C127 |
| Cambodia | KH180 | 2013 | Kampot | N/A | 1.29 | N/A | N/A | C127 |
| Cambodia | KH181 | 2013 | Kampot | N/A | 1.46 | N/A | N/A | C127 |
| Cambodia | KH182 | 2013 | Kampot | N/A | 1.42 | 3 | N/A | N/A |
| Cambodia | KH183 | 2013 | Pailin | N/A | 0.57 | 14 | N/A | N/A |
| Cambodia | KH184 | 2013 | Pailin | N/A | 1.22 | 2 | 0.84 | 1 |
| Cambodia | KH185 | 2013 | Kampot | TCT | 0.93 | 14 | 1.11 | 1 |
| Cambodia | KH186 | 2013 | Pailin | N/A | 1.00 | 1 | 0.78 | 2 |
| Cambodia | KH187 | 2013 | Kampot | N/A | 0.74 | 9 | 1.24 | 3 |
| Cambodia | KH188 | 2013 | Pailin | N/A | 1.36 | 6 | 1.44 | 6 |
| Cambodia | KH189 | 2013 | Kampot | TCT | 1.37 | 7 | 0.83 | 6 |
| Cambodia | KH190 | 2013 | Pailin | N/A | 1.36 | 9 | 1.42 | 6 |
| Cambodia | KH191 | 2013 | Kampot | TCT | 1.05 | 1 | 1.06 | C127 |
| Cambodia | KH192 | 2013 | Kampot | N/A | 0.88 | 3 | 1.07 | C127 |
| Cambodia | KH193 | 2013 | Kampot | N/A | 1.02 | 3 | 1.01 | C127 |
| Cambodia | KH194 | 2013 | Kampot | N/A | 1.18 | 3 | 1.16 | C127 |
| Cambodia | KH195 | 2013 | Pailin | N/A | 1.22 | 3 | 0.88 | C127 |
| Cambodia | KH196 | 2013 | Kampot | TCT | 1.33 | 3 | 1.27 | C127 |
| Cambodia | KH197 | 2013 | Pailin | N/A | 0.85 | 4 | 0.70 | C127 |
| Cambodia | KH198 | 2013 | Pailin | N/A | 1.26 | 4 | 0.51 | C127 |
| Cambodia | KH199 | 2013 | Pailin | N/A | 0.80 | 5 | 1.34 | C127 |
| Cambodia | KH200 | 2013 | Kampot | N/A | 0.84 | 6 | 0.53 | C127 |
| Cambodia | KH201 | 2013 | Pailin | N/A | 1.07 | 6 | 0.71 | C127 |
| Cambodia | KH202 | 2013 | Kampot | TCT | 1.08 | 6 | 1.29 | C127 |
| Cambodia | KH203 | 2013 | Kampot | N/A | 0.81 | 7 | 1.11 | C127 |
| Cambodia | KH204 | 2013 | Pailin | N/A | 1.37 | 7 | 1.32 | C127 |
| Cambodia | KH205 | 2013 | Kampot | N/A | 1.16 | 8 | 0.90 | C127 |
| Cambodia | KH206 | 2013 | Kampot | N/A | 1.30 | 8 | 0.74 | C127 |
| Cambodia | KH207 | 2013 | Pailin | N/A | 1.19 | 10 | 1.16 | C127 |
| Cambodia | KH208 | 2013 | Pursat | N/A | 1.36 | 10 | 0.75 | C127 |
| Cambodia | KH209 | 2013 | Kampot | N/A | 1.19 | 12 | 1.07 | C127 |
| Cambodia | KH210 | 2013 | Kampot | N/A | 0.68 | 14 | 1.09 | C127 |
| Cambodia | KH211 | 2013 | Kratie | N/A | 1.47 | 27 | 0.80 | C127 |
| Cambodia | KH212 | 2013 | Kampot | N/A | 0.67 | 2 | 2.32 | 6 |
| Cambodia | KH213 | 2013 | Kampot | TCT | 0.98 | Sal1 | 2.22 | 6 |
| Cambodia | KH214 | 2013 | Kampot | TCT | 1.32 | 10 | 1.96 | C127 |
| Cambodia | KH215 | 2013 | Pailin | N/A | 2.95 | 7 | N/A | 5 |
| Cambodia | KH216 | 2013 | Pailin | N/A | 2.28 | 3 | N/A | C127 |
| Cambodia | KH217 | 2013 | Kampot | N/A | 2.71 | 9 | N/A | C127 |
| Cambodia | KH218 | 2013 | Phnom Penh | N/A | 1.61 | Sal1 | N/A | C127 |
| Cambodia | KH219 | 2013 | Kampot | TCT | 1.52 | N/A | N/A | C127 |
| Cambodia | KH220 | 2013 | Kratie | N/A | 1.84 | N/A | N/A | C127 |
| Cambodia | KH221 | 2013 | Pailin | N/A | 2.12 | N/A | N/A | C127 |
| Cambodia | KH222 | 2013 | Kampot | N/A | 1.86 | 2 | N/A | N/A |
| Cambodia | KH223 | 2013 | Kampot | TCT | 1.88 | 3 | 1.29 | 2 |
| Cambodia | KH224 | 2013 | Kratie | N/A | 2.71 | 3 | 0.77 | 3 |
| Cambodia | KH225 | 2013 | Kampot | N/A | 1.60 | 3 | 1.00 | 6 |
| Cambodia | KH226 | 2013 | Pailin | N/A | 3.47 | 6 | 1.45 | 6 |
| Cambodia | KH227 | 2013 | Pailin | N/A | 1.67 | 9 | 1.24 | 6 |
| Cambodia | KH228 | 2013 | Kampot | N/A | 5.06 | 7 | 0.75 | 7 |
| Cambodia | KH229 | 2013 | Kampot | N/A | 1.94 | 1 | 1.21 | C127 |
| Cambodia | KH230 | 2013 | Kampot | TCT | 1.62 | 2 | 1.40 | C127 |
| Cambodia | KH231 | 2013 | Pailin | N/A | 1.54 | 3 | 0.93 | C127 |
| Cambodia | KH232 | 2013 | Kampot | N/A | 2.45 | 3 | 1.37 | C127 |
| Cambodia | KH233 | 2013 | Kampot | TCT | 3.93 | 3 | 1.26 | C127 |
| Cambodia | KH234 | 2013 | Pailin | N/A | 1.58 | 6 | 0.53 | C127 |
| Cambodia | KH235 | 2013 | Pailin | N/A | 2.45 | 7 | 1.28 | C127 |
| Cambodia | KH236 | 2013 | Pailin | N/A | 3.24 | 7 | 1.01 | C127 |
| Cambodia | KH237 | 2013 | Kampot | TCT | 2.05 | 9 | 0.97 | C127 |
| Cambodia | KH238 | 2013 | Pailin | N/A | 1.55 | 10 | 0.82 | C127 |
| Cambodia | KH239 | 2013 | Kampot | TCT | 1.55 | 2 | 1.65 | 4 |
| Cambodia | KH240 | 2014 | Kampot | N/A | N/A | N/A | N/A | C127 |
| Cambodia | KH241 | 2014 | Kampot | N/A | N/A | N/A | N/A | C127 |
| Cambodia | KH242 | 2014 | Kampot | N/A | N/A | N/A | N/A | C127 |
| Cambodia | KH243 | 2014 | Ratanakiri | N/A | 0.50 | N/A | N/A | N/A |
| Cambodia | KH244 | 2014 | Ratanakiri | N/A | 0.57 | N/A | N/A | N/A |
| Cambodia | KH245 | 2014 | Ratanakiri | N/A | 0.64 | N/A | N/A | N/A |
| Cambodia | KH246 | 2014 | Ratanakiri | N/A | 0.64 | N/A | N/A | N/A |
| Cambodia | KH247 | 2014 | Ratanakiri | N/A | 0.67 | N/A | N/A | N/A |
| Cambodia | KH248 | 2014 | Ratanakiri | N/A | 0.67 | N/A | N/A | N/A |
| Cambodia | KH249 | 2014 | Ratanakiri | N/A | 0.72 | N/A | N/A | N/A |
| Cambodia | KH250 | 2014 | Ratanakiri | N/A | 0.80 | N/A | N/A | N/A |
| Cambodia | KH251 | 2014 | Ratanakiri | N/A | 0.90 | N/A | N/A | N/A |
| Cambodia | KH252 | 2014 | Ratanakiri | N/A | 0.93 | N/A | N/A | N/A |
| Cambodia | KH253 | 2014 | Ratanakiri | N/A | 0.94 | N/A | N/A | N/A |
| Cambodia | KH254 | 2014 | Ratanakiri | N/A | 0.97 | N/A | N/A | N/A |
| Cambodia | KH255 | 2014 | Ratanakiri | N/A | 0.97 | N/A | N/A | N/A |
| Cambodia | KH256 | 2014 | Ratanakiri | N/A | 1.00 | N/A | N/A | N/A |
| Cambodia | KH257 | 2014 | Ratanakiri | N/A | 1.05 | N/A | N/A | N/A |
| Cambodia | KH258 | 2014 | Ratanakiri | N/A | 1.06 | N/A | N/A | N/A |
| Cambodia | KH259 | 2014 | Ratanakiri | N/A | 1.06 | N/A | N/A | N/A |
| Cambodia | KH260 | 2014 | Ratanakiri | N/A | 1.07 | N/A | N/A | N/A |
| Cambodia | KH261 | 2014 | Ratanakiri | N/A | 1.10 | N/A | N/A | N/A |
| Cambodia | KH262 | 2014 | Ratanakiri | N/A | 1.14 | N/A | N/A | N/A |
| Cambodia | KH263 | 2014 | Ratanakiri | N/A | 1.17 | N/A | N/A | N/A |
| Cambodia | KH264 | 2014 | Ratanakiri | N/A | 1.19 | N/A | N/A | N/A |
| Cambodia | KH265 | 2014 | Ratanakiri | N/A | 1.20 | N/A | N/A | N/A |
| Cambodia | KH266 | 2014 | Ratanakiri | TCT | 1.20 | N/A | N/A | N/A |
| Cambodia | KH267 | 2014 | Ratanakiri | N/A | 1.20 | N/A | N/A | N/A |
| Cambodia | KH268 | 2014 | Ratanakiri | N/A | 1.21 | N/A | N/A | N/A |
| Cambodia | KH269 | 2014 | Ratanakiri | N/A | 1.26 | N/A | N/A | N/A |
| Cambodia | KH270 | 2014 | Ratanakiri | N/A | N/A | N/A | 1.42 | N/A |
| Cambodia | KH271 | 2014 | Ratanakiri | N/A | N/A | N/A | 0.60 | N/A |
| Cambodia | KH272 | 2014 | Ratanakiri | N/A | 1.30 | N/A | N/A | N/A |
| Cambodia | KH273 | 2014 | Ratanakiri | N/A | 1.30 | N/A | N/A | N/A |
| Cambodia | KH274 | 2014 | Ratanakiri | N/A | 1.32 | N/A | N/A | N/A |
| Cambodia | KH275 | 2014 | Ratanakiri | N/A | 1.37 | N/A | N/A | N/A |
| Cambodia | KH276 | 2014 | Ratanakiri | N/A | 1.37 | N/A | N/A | N/A |
| Cambodia | KH277 | 2014 | Ratanakiri | N/A | 1.38 | N/A | N/A | N/A |
| Cambodia | KH278 | 2014 | Ratanakiri | N/A | 1.47 | N/A | N/A | N/A |
| Cambodia | KH279 | 2014 | Ratanakiri | N/A | 1.02 | N/A | N/A | N/A |
| Cambodia | KH280 | 2014 | Ratanakiri | N/A | 0.91 | N/A | N/A | N/A |
| Cambodia | KH281 | 2014 | Ratanakiri | N/A | 0.99 | N/A | N/A | N/A |
| Cambodia | KH282 | 2014 | Ratanakiri | N/A | 1.27 | N/A | N/A | N/A |
| Cambodia | KH283 | 2014 | Ratanakiri | N/A | 1.02 | N/A | N/A | N/A |
| Cambodia | KH284 | 2014 | Ratanakiri | N/A | 1.47 | N/A | N/A | N/A |
| Cambodia | KH285 | 2014 | Ratanakiri | N/A | 1.23 | N/A | N/A | N/A |
| Cambodia | KH286 | 2014 | Ratanakiri | N/A | 0.59 | N/A | N/A | N/A |
| Cambodia | KH287 | 2014 | Ratanakiri | N/A | N/A | N/A | 1.18 | N/A |
| Cambodia | KH288 | 2014 | Ratanakiri | N/A | N/A | N/A | 0.75 | N/A |
| Cambodia | KH289 | 2014 | Ratanakiri | N/A | N/A | N/A | 1.45 | N/A |
| Cambodia | KH290 | 2014 | Ratanakiri | N/A | N/A | N/A | 1.45 | N/A |
| Cambodia | KH291 | 2014 | Ratanakiri | N/A | N/A | N/A | 0.86 | N/A |
| Cambodia | KH292 | 2014 | Ratanakiri | TCT | 1.28 | 2 | 1.32 | N/A |
| Cambodia | KH293 | 2014 | Ratanakiri | N/A | 1.19 | 3 | 0.81 | N/A |
| Cambodia | KH294 | 2014 | Ratanakiri | N/A | 0.70 | 12 | 1.27 | N/A |
| Cambodia | KH295 | 2014 | Ratanakiri | N/A | 0.63 | N/A | 1.10 | N/A |
| Cambodia | KH296 | 2014 | Ratanakiri | N/A | 0.70 | N/A | 0.69 | N/A |
| Cambodia | KH297 | 2014 | Ratanakiri | N/A | 0.72 | N/A | 0.73 | N/A |
| Cambodia | KH298 | 2014 | Ratanakiri | N/A | 0.79 | N/A | 1.06 | N/A |
| Cambodia | KH299 | 2014 | Ratanakiri | N/A | 0.85 | N/A | 1.42 | N/A |
| Cambodia | KH300 | 2014 | Ratanakiri | N/A | 1.14 | N/A | 0.98 | N/A |
| Cambodia | KH301 | 2014 | Ratanakiri | N/A | 1.00 | N/A | 0.60 | N/A |
| Cambodia | KH302 | 2014 | Ratanakiri | N/A | 1.43 | N/A | 0.66 | N/A |
| Cambodia | KH303 | 2014 | Ratanakiri | N/A | 0.55 | N/A | 2.10 | 6 |
| Cambodia | KH304 | 2014 | Ratanakiri | N/A | 0.80 | N/A | 1.87 | N/A |
| Cambodia | KH305 | 2014 | Ratanakiri | N/A | 0.93 | N/A | 1.71 | N/A |
| Cambodia | KH306 | 2014 | Ratanakiri | N/A | 1.25 | N/A | 1.55 | N/A |
| Cambodia | KH307 | 2014 | Ratanakiri | N/A | 0.91 | N/A | 2.10 | N/A |
| Cambodia | KH308 | 2014 | Ratanakiri | N/A | 1.39 | N/A | 1.73 | N/A |
| Cambodia | KH309 | 2014 | Ratanakiri | N/A | 1.42 | N/A | 1.72 | N/A |
| Cambodia | KH310 | 2014 | Ratanakiri | N/A | 1.49 | N/A | 1.66 | N/A |
| Cambodia | KH311 | 2014 | Pursat | N/A | 1.94 | N/A | N/A | 4 |
| Cambodia | KH312 | 2014 | Ratanakiri | TCT | 3.04 | 2 | N/A | 5 |
| Cambodia | KH313 | 2014 | Ratanakiri | TCT | 3.22 | 7 | N/A | 6 |
| Cambodia | KH314 | 2014 | Ratanakiri | TCT | 2.68 | 9 | N/A | 6 |
| Cambodia | KH315 | 2014 | Ratanakiri | TCT | 4.24 | 1 | N/A | C127 |
| Cambodia | KH316 | 2014 | Ratanakiri | TCT | 1.84 | 3 | N/A | C127 |
| Cambodia | KH317 | 2014 | Ratanakiri | TCT | 6.17 | 7 | N/A | C127 |
| Cambodia | KH318 | 2014 | Ratanakiri | TCT | 2.13 | 9 | N/A | N/A |
| Cambodia | KH319 | 2014 | Ratanakiri | N/A | N/A | N/A | 1.90 | N/A |
| Cambodia | KH320 | 2014 | Ratanakiri | N/A | 1.53 | N/A | N/A | N/A |
| Cambodia | KH321 | 2014 | Ratanakiri | N/A | 1.60 | N/A | N/A | N/A |
| Cambodia | KH322 | 2014 | Ratanakiri | N/A | 3.68 | N/A | N/A | N/A |
| Cambodia | KH323 | 2014 | Ratanakiri | N/A | 3.83 | N/A | N/A | N/A |
| Cambodia | KH324 | 2014 | Ratanakiri | N/A | 1.90 | N/A | N/A | N/A |
| Cambodia | KH325 | 2014 | Ratanakiri | N/A | 1.90 | N/A | N/A | N/A |
| Cambodia | KH326 | 2014 | Ratanakiri | N/A | 1.95 | N/A | N/A | N/A |
| Cambodia | KH327 | 2014 | Ratanakiri | N/A | 2.12 | N/A | N/A | N/A |
| Cambodia | KH328 | 2014 | Ratanakiri | N/A | 2.12 | N/A | N/A | N/A |
| Cambodia | KH329 | 2014 | Ratanakiri | N/A | 2.14 | N/A | N/A | N/A |
| Cambodia | KH330 | 2014 | Ratanakiri | N/A | 2.18 | N/A | N/A | N/A |
| Cambodia | KH331 | 2014 | Ratanakiri | N/A | 1.74 | N/A | N/A | N/A |
| Cambodia | KH332 | 2014 | Ratanakiri | N/A | 2.26 | N/A | N/A | N/A |
| Cambodia | KH333 | 2014 | Ratanakiri | N/A | 2.34 | N/A | N/A | N/A |
| Cambodia | KH334 | 2014 | Ratanakiri | N/A | 2.49 | N/A | N/A | N/A |
| Cambodia | KH335 | 2014 | Ratanakiri | N/A | 2.50 | N/A | N/A | N/A |
| Cambodia | KH336 | 2014 | Ratanakiri | N/A | 2.74 | N/A | N/A | N/A |
| Cambodia | KH337 | 2014 | Ratanakiri | N/A | 3.07 | N/A | N/A | N/A |
| Cambodia | KH338 | 2014 | Ratanakiri | N/A | 3.34 | N/A | N/A | N/A |
| Cambodia | KH339 | 2014 | Ratanakiri | N/A | 3.67 | N/A | N/A | N/A |
| Cambodia | KH340 | 2014 | Ratanakiri | N/A | 4.00 | N/A | N/A | N/A |
| Cambodia | KH341 | 2014 | Ratanakiri | N/A | 4.71 | N/A | N/A | N/A |
| Cambodia | KH342 | 2014 | Ratanakiri | N/A | N/A | N/A | 1.63 | N/A |
| Cambodia | KH343 | 2014 | Ratanakiri | N/A | N/A | N/A | 1.51 | N/A |
| Cambodia | KH344 | 2014 | Ratanakiri | N/A | N/A | N/A | 1.55 | N/A |
| Cambodia | KH345 | 2014 | Ratanakiri | N/A | N/A | N/A | 1.72 | N/A |
| Cambodia | KH346 | 2014 | Ratanakiri | TCT | 1.67 | 28 | 0.86 | 3 |
| Cambodia | KH347 | 2014 | Ratanakiri | TCT | 3.32 | 3 | 0.65 | 6 |
| Cambodia | KH348 | 2014 | Ratanakiri | N/A | 5.19 | 3 | 0.85 | 6 |
| Cambodia | KH349 | 2014 | Ratanakiri | TCT | 1.53 | 9 | 1.41 | 6 |
| Cambodia | KH350 | 2014 | Ratanakiri | TCT | 1.96 | 3 | 0.97 | C127 |
| Cambodia | KH351 | 2014 | Ratanakiri | TCT | 1.74 | 3 | 0.87 | N/A |
| Cambodia | KH352 | 2014 | Ratanakiri | N/A | 1.82 | N/A | 1.36 | N/A |
| Cambodia | KH353 | 2014 | Ratanakiri | N/A | 2.32 | N/A | 0.78 | N/A |
| Cambodia | KH354 | 2014 | Ratanakiri | TCT | 2.54 | N/A | 1.31 | N/A |
| Cambodia | KH355 | 2014 | Ratanakiri | TCT | 2.56 | N/A | 0.97 | N/A |
| Cambodia | KH356 | 2014 | Ratanakiri | N/A | 4.22 | N/A | 1.25 | N/A |
| Cambodia | KH357 | 2014 | Ratanakiri | TCT | 2.70 | 3 | 1.73 | 10 |
| Cambodia | KH358 | 2014 | Ratanakiri | N/A | 3.73 | 3 | 1.52 | N/A |
| Cambodia | KH359 | 2014 | Ratanakiri | N/A | 1.64 | N/A | 1.66 | N/A |
| Cambodia | KH360 | 2014 | Ratanakiri | N/A | 3.25 | N/A | 1.61 | N/A |
| Cambodia | KH361 | 2014 | Ratanakiri | N/A | 3.93 | N/A | 1.60 | N/A |
| Cambodia | KH362 | 2015 | Kampot | N/A | N/A | N/A | N/A | C127 |
| Cambodia | KH363 | 2015 | Ratanakiri | TCT | N/A | 2 | 1.01 | 1 |
| Cambodia | KH364 | 2015 | Ratanakiri | TCT | N/A | 2 | 0.94 | 2 |
| Cambodia | KH365 | 2015 | Ratanakiri | TCT | N/A | 6 | 0.75 | 5 |
| Cambodia | KH366 | 2015 | Ratanakiri | TCT | N/A | 7 | 1.08 | 6 |
| Cambodia | KH367 | 2015 | Ratanakiri | TCT | N/A | 30 | 0.87 | 6 |
| Cambodia | KH368 | 2015 | Ratanakiri | TCT | N/A | 3 | 0.99 | C127 |
| Cambodia | KH369 | 2015 | Ratanakiri | TCT | N/A | 3 | 0.80 | C127 |
| Cambodia | KH370 | 2015 | Ratanakiri | TCT | N/A | 32 | 0.70 | C127 |
| Cambodia | KH371 | 2015 | Ratanakiri | TCT | N/A | N/A | 1.33 | C127 |
| Cambodia | KH372 | 2015 | Ratanakiri | TCT | N/A | 9 | 1.15 | 11 |
| Cambodia | KH373 | 2015 | Ratanakiri | TCT | 0.58 | 6 | N/A | N/A |
| Cambodia | KH374 | 2015 | Ratanakiri | TCT | 0.74 | 6 | N/A | N/A |
| Cambodia | KH375 | 2015 | Ratanakiri | TCT | N/A | 10 | 0.61 | N/A |
| Cambodia | KH376 | 2015 | Ratanakiri | TCT | 0.87 | 29 | N/A | N/A |
| Cambodia | KH377 | 2015 | Ratanakiri | TCT | 1.20 | N/A | N/A | N/A |
| Cambodia | KH378 | 2015 | Ratanakiri | TCT | 1.10 | 7 | 1.07 | 6 |
| Cambodia | KH379 | 2015 | Ratanakiri | TCT | 0.92 | 9 | 1.15 | 6 |
| Cambodia | KH380 | 2015 | Ratanakiri | TCT | 0.60 | 11 | 1.06 | 6 |
| Cambodia | KH381 | 2015 | Ratanakiri | TCT | 1.18 | 3 | 1.46 | C127 |
| Cambodia | KH382 | 2015 | Ratanakiri | TCT | 0.86 | 6 | 1.03 | C127 |
| Cambodia | KH383 | 2015 | Ratanakiri | TCT | 0.88 | 7 | 1.40 | C127 |
| Cambodia | KH384 | 2015 | Ratanakiri | TCT | 1.07 | 10 | 1.09 | C127 |
| Cambodia | KH385 | 2015 | Ratanakiri | TCT | 0.61 | 12 | 1.30 | C127 |
| Cambodia | KH386 | 2015 | Ratanakiri | TCT | 0.98 | 3 | 1.34 | 11 |
| Cambodia | KH387 | 2015 | Ratanakiri | TCT | 0.84 | 12 | 1.14 | N/A |
| Cambodia | KH388 | 2015 | Ratanakiri | TCT | 1.06 | N/A | 1.34 | N/A |
| Cambodia | KH389 | 2015 | Ratanakiri | TCT | 1.10 | N/A | 0.82 | N/A |
| Cambodia | KH390 | 2015 | Ratanakiri | TCT | 1.19 | N/A | 1.44 | N/A |
| Cambodia | KH391 | 2015 | Ratanakiri | TCT | 1.00 | 10 | 2.32 | 5 |
| Cambodia | KH392 | 2015 | Ratanakiri | TCT | 0.87 | 3 | 1.83 | C127 |
| Cambodia | KH393 | 2015 | Ratanakiri | TCT | 0.81 | 6 | 2.05 | C127 |
| Cambodia | KH394 | 2015 | Ratanakiri | TCT | 0.84 | 6 | 1.50 | N/A |
| Cambodia | KH395 | 2015 | Ratanakiri | TCT | 1.26 | 26 | 1.77 | N/A |
| Cambodia | KH396 | 2015 | Ratanakiri | TCT | N/A | 3 | 1.82 | C127 |
| Cambodia | KH397 | 2015 | Ratanakiri | TCT | 2.48 | 3 | N/A | N/A |
| Cambodia | KH398 | 2015 | Ratanakiri | TCT | 3.47 | 3 | N/A | N/A |
| Cambodia | KH399 | 2015 | Ratanakiri | TCT | 1.93 | 3 | 0.74 | 4 |
| Cambodia | KH400 | 2015 | Ratanakiri | TCT | 2.20 | 2 | 1.26 | 5 |
| Cambodia | KH401 | 2015 | Ratanakiri | TCT | 2.51 | 1 | 1.44 | 6 |
| Cambodia | KH402 | 2015 | Ratanakiri | TCT | 1.76 | 7 | 1.38 | 6 |
| Cambodia | KH403 | 2015 | Ratanakiri | TCT | 1.77 | 9 | 0.90 | 6 |
| Cambodia | KH404 | 2015 | Ratanakiri | TCT | 2.87 | 3 | 1.36 | C127 |
| Cambodia | KH405 | 2015 | Ratanakiri | TCT | 1.52 | 7 | 1.30 | C127 |
| Cambodia | KH406 | 2015 | Ratanakiri | TCT | 1.88 | 8 | 1.28 | C127 |
| Cambodia | KH407 | 2015 | Ratanakiri | TCT | 1.62 | 3 | 0.84 | N/A |
| Cambodia | KH408 | 2015 | Ratanakiri | TCT | 2.04 | 3 | 0.53 | N/A |
| Cambodia | KH409 | 2015 | Ratanakiri | TCT | 2.09 | 9 | 1.31 | N/A |
| Cambodia | KH410 | 2015 | Ratanakiri | TCT | 3.36 | 3 | 2.04 | 2 |
| Cambodia | KH411 | 2015 | Ratanakiri | TCT | 1.57 | 6 | 1.92 | 6 |
| Cambodia | KH412 | 2015 | Ratanakiri | TCT | 1.84 | 3 | 2.31 | C127 |
| Cambodia | KH413 | 2015 | Ratanakiri | TCT | 1.80 | 9 | 1.96 | C127 |
| Cambodia | KH414 | 2015 | Ratanakiri | TCT | 1.95 | N/A | 1.87 | N/A |
| Cambodia | KH415 | 2015 | Ratanakiri | TCT | 2.65 | N/A | 1.87 | N/A |
| Cambodia | KH416 | 2016 | Ratanakiri | N/A | N/A | N/A | N/A | C127 |
| Cambodia | KH417 | 2016 | Ratanakiri | N/A | 1.29 | N/A | N/A | N/A |
| Cambodia | KH418 | 2016 | Ratanakiri | TCT | 0.61 | 6 | 1.40 | 2 |
| Cambodia | KH419 | 2016 | Ratanakiri | TCT | 0.90 | 6 | 1.13 | 4 |
| Cambodia | KH420 | 2016 | Ratanakiri | TCT | 0.84 | 2 | 1.05 | C127 |
| Cambodia | KH421 | 2016 | Ratanakiri | TCT | 0.69 | 3 | 1.48 | C127 |
| Cambodia | KH422 | 2016 | Ratanakiri | TCT | 1.38 | 3 | 0.72 | C127 |
| Cambodia | KH423 | 2016 | Ratanakiri | TCT | 0.52 | 4 | 1.37 | C127 |
| Cambodia | KH424 | 2016 | Ratanakiri | TCT | 0.79 | 32 | 1.32 | 10 |
| Cambodia | KH425 | 2016 | Ratanakiri | TCT | 0.72 | N/A | 1.48 | N/A |
| Cambodia | KH426 | 2016 | Ratanakiri | TCT | 0.77 | N/A | 1.44 | N/A |
| Cambodia | KH427 | 2016 | Ratanakiri | TCT | 0.77 | N/A | 1.29 | N/A |
| Cambodia | KH428 | 2016 | Ratanakiri | TCT | 0.80 | N/A | 1.47 | N/A |
| Cambodia | KH429 | 2016 | Ratanakiri | TCT | 0.57 | N/A | 1.84 | N/A |
| Cambodia | KH430 | 2016 | Ratanakiri | TCT | 1.45 | N/A | 1.92 | N/A |
| Cambodia | KH431 | 2016 | Ratanakiri | TCT | 2.81 | 1 | 1.30 | 5 |
| Cambodia | KH432 | 2016 | Ratanakiri | TCT | 3.23 | 31 | 1.34 | C127 |
| Cambodia | KH433 | 2016 | Ratanakiri | TCT | 1.54 | N/A | 1.51 | N/A |
| Cambodia | KH434 | 2017 | Mondulkiri | TCT | 0.65 | N/A | 1.32 | N/A |
| Cambodia | KH435 | 2017 | Mondulkiri | TCT | 0.87 | N/A | 1.09 | N/A |
| Cambodia | KH436 | 2017 | Mondulkiri | TCT | 0.92 | N/A | 1.18 | N/A |
| Cambodia | KH437 | 2017 | Mondulkiri | TCT | 0.95 | N/A | 1.37 | N/A |
| Cambodia | KH438 | 2017 | Mondulkiri | TCT | 0.98 | N/A | 1.30 | N/A |
| Cambodia | KH439 | 2017 | Mondulkiri | TCT | 1.04 | N/A | 1.02 | N/A |
| Cambodia | KH440 | 2017 | Mondulkiri | TCT | 1.07 | N/A | 1.23 | N/A |
| Cambodia | KH441 | 2017 | Mondulkiri | TCT | 1.08 | N/A | 0.84 | N/A |
| Cambodia | KH442 | 2017 | Mondulkiri | TCT | 1.10 | N/A | 0.82 | N/A |
| Cambodia | KH443 | 2017 | Mondulkiri | TCT | 1.15 | N/A | 1.34 | N/A |
| Cambodia | KH444 | 2017 | Mondulkiri | TCT | 1.57 | N/A | 1.16 | N/A |
| Cambodia | KH445 | 2017 | Mondulkiri | TCT | 1.63 | N/A | 1.21 | N/A |
| Cambodia | KH446 | 2017 | Mondulkiri | TCT | 1.74 | N/A | 1.27 | N/A |
| Cambodia | KH447 | 2017 | Mondulkiri | TCT | 1.84 | N/A | 1.28 | N/A |
| Cambodia | KH448 | 2017 | Mondulkiri | TCT | 1.88 | N/A | 1.44 | N/A |
| Cambodia | KH449 | 2017 | Mondulkiri | TCT | 1.91 | N/A | 1.10 | N/A |
| Cambodia | KH450 | 2017 | Mondulkiri | TCT | 2.05 | N/A | 1.32 | N/A |
| Cambodia | KH451 | 2017 | Mondulkiri | TCT | 2.20 | N/A | 1.44 | N/A |
| Cambodia | KH452 | 2017 | Mondulkiri | TCT | 2.48 | N/A | 1.31 | N/A |
| Cambodia | KH453 | 2017 | Mondulkiri | TCT | 2.78 | N/A | 0.85 | N/A |
| Madagascar | MAD001 | 2015 | Maevatanana | N/A | N/A | N/A | N/A | 4 |
| Madagascar | MAD002 | 2015 | Maevatanana | N/A | N/A | N/A | N/A | 5 |
| Madagascar | MAD003 | 2015 | Maevatanana | N/A | N/A | 13 | N/A | C127 |
| Madagascar | MAD004 | 2015 | Maevatanana | N/A | N/A | 14 | N/A | C127 |
| Madagascar | MAD005 | 2015 | Maevatanana | N/A | N/A | N/A | N/A | C127 |
| Madagascar | MAD006 | 2015 | Maevatanana | N/A | N/A | N/A | N/A | C127 |
| Madagascar | MAD007 | 2015 | Maevatanana | N/A | N/A | N/A | N/A | C127 |
| Madagascar | MAD008 | 2015 | Maevatanana | N/A | N/A | N/A | N/A | C127 |
| Madagascar | MAD009 | 2015 | Maevatanana | N/A | N/A | N/A | N/A | C127 |
| Madagascar | MAD010 | 2015 | Maevatanana | N/A | N/A | N/A | N/A | C127 |
| Madagascar | MAD011 | 2015 | Maevatanana | N/A | N/A | N/A | N/A | C127 |
| Madagascar | MAD012 | 2015 | Maevatanana | N/A | N/A | N/A | N/A | C127 |
| Madagascar | MAD013 | 2015 | Maevatanana | N/A | N/A | 13 | N/A | N/A |
| Madagascar | MAD014 | 2015 | Maevatanana | N/A | N/A | 13 | N/A | N/A |
| Madagascar | MAD015 | 2015 | Maevatanana | N/A | N/A | 14 | N/A | N/A |
| Madagascar | MAD016 | 2015 | Maevatanana | N/A | N/A | 14 | N/A | N/A |
| Madagascar | MAD017 | 2015 | Maevatanana | N/A | N/A | 16 | N/A | N/A |
| Madagascar | MAD018 | 2015 | Maevatanana | TCT | 1.02 | N/A | N/A | 5 |
| Madagascar | MAD019 | 2015 | Maevatanana | TCT | 1.31 | 14 | 1.02 | 5 |
| Madagascar | MAD020 | 2015 | Maevatanana | TCT/CCT | 1.18 | 10 | 1.42 | N/A |
| Madagascar | MAD021 | 2015 | Maevatanana | TCT/CCT | 1.19 | N/A | 1.19 | N/A |
| Madagascar | MAD022 | 2015 | Maevatanana | TCT | 1.40 | N/A | 2.22 | 6 |
| Madagascar | MAD023 | 2015 | Maevatanana | TCT | 0.90 | N/A | 4.27 | C127 |
| Madagascar | MAD024 | 2015 | Maevatanana | TCT | 1.12 | 10 | 2.26 | N/A |
| Madagascar | MAD025 | 2015 | Maevatanana | TCT/CCT | 1.18 | 14 | 2.15 | N/A |
| Madagascar | MAD026 | 2015 | Maevatanana | TCT/CCT | 1.35 | 14 | 1.85 | N/A |
| Madagascar | MAD027 | 2015 | Maevatanana | TCT | 0.96 | N/A | 1.69 | N/A |
| Madagascar | MAD028 | 2015 | Maevatanana | TCT | N/A | 14 | 1.58 | C127 |
| Madagascar | MAD029 | 2015 | Maevatanana | TCT/CCT | 1.94 | 22 | N/A | C127 |
| Madagascar | MAD030 | 2015 | Maevatanana | TCT | N/A | 13 | 3.64 | 9 |
| Madagascar | MAD031 | 2015 | Maevatanana | TCT | 3.66 | 19 | N/A | N/A |
| Madagascar | MAD032 | 2015 | Maevatanana | TCT/CCT | 3.65 | N/A | N/A | N/A |
| Madagascar | MAD033 | 2015 | Maevatanana | TCT | 3.44 | N/A | 0.94 | 2 |
| Madagascar | MAD034 | 2015 | Maevatanana | TCT | 2.36 | N/A | 1.26 | C127 |
| Madagascar | MAD035 | 2015 | Maevatanana | TCT/CCT | 1.63 | Sal1 | 1.29 | 9 |
| Madagascar | MAD036 | 2015 | Maevatanana | TCT | 1.68 | 17 | 1.36 | 9 |
| Madagascar | MAD037 | 2015 | Maevatanana | TCT | 2.50 | 14 | 1.45 | N/A |
| Madagascar | MAD038 | 2015 | Maevatanana | TCT | 3.52 | 10 | 1.62 | C127 |
| Madagascar | MAD039 | 2015 | Maevatanana | TCT | 3.64 | 17 | 2.63 | C127 |
| Madagascar | MAD040 | 2015 | Maevatanana | TCT | 4.37 | 17 | 1.51 | C127 |
| Madagascar | MAD041 | 2015 | Maevatanana | TCT | 2.33 | N/A | 2.40 | C127 |
| Madagascar | MAD042 | 2015 | Maevatanana | TCT | 2.08 | 10 | 2.00 | N/A |
| Madagascar | MAD043 | 2015 | Maevatanana | TCT | 3.02 | 20 | 2.02 | N/A |
| Madagascar | MAD044 | 2015 | Maevatanana | TCT/CCT | 1.87 | 22 | 1.68 | N/A |
| Madagascar | MAD045 | 2016 | Maevatanana | N/A | N/A | 10 | N/A | 4 |
| Madagascar | MAD046 | 2016 | Maevatanana | N/A | N/A | Sal1 | N/A | 4 |
| Madagascar | MAD047 | 2016 | Maevatanana | N/A | N/A | 10 | N/A | 5 |
| Madagascar | MAD048 | 2016 | Maevatanana | N/A | N/A | 14 | N/A | 6 |
| Madagascar | MAD049 | 2016 | Maevatanana | N/A | N/A | 10 | N/A | C127 |
| Madagascar | MAD050 | 2016 | Maevatanana | N/A | N/A | 10 | N/A | C127 |
| Madagascar | MAD051 | 2016 | Maevatanana | N/A | N/A | 10 | N/A | C127 |
| Madagascar | MAD052 | 2016 | Maevatanana | N/A | N/A | 10 | N/A | C127 |
| Madagascar | MAD053 | 2016 | Maevatanana | N/A | N/A | 13 | N/A | C127 |
| Madagascar | MAD054 | 2016 | Maevatanana | N/A | N/A | 13 | N/A | C127 |
| Madagascar | MAD055 | 2016 | Maevatanana | N/A | N/A | 14 | N/A | C127 |
| Madagascar | MAD056 | 2016 | Maevatanana | N/A | N/A | 14 | N/A | C127 |
| Madagascar | MAD057 | 2016 | Maevatanana | N/A | N/A | 15 | N/A | C127 |
| Madagascar | MAD058 | 2016 | Maevatanana | N/A | N/A | 17 | N/A | C127 |
| Madagascar | MAD059 | 2016 | Maevatanana | N/A | N/A | 17 | N/A | C127 |
| Madagascar | MAD060 | 2016 | Maevatanana | N/A | N/A | N/A | N/A | C127 |
| Madagascar | MAD061 | 2016 | Maevatanana | N/A | N/A | N/A | N/A | C127 |
| Madagascar | MAD062 | 2016 | Maevatanana | N/A | N/A | 13 | N/A | 8 |
| Madagascar | MAD063 | 2016 | Maevatanana | N/A | N/A | 13 | N/A | 9 |
| Madagascar | MAD064 | 2016 | Maevatanana | N/A | N/A | 16 | N/A | 9 |
| Madagascar | MAD065 | 2016 | Maevatanana | N/A | N/A | N/A | N/A | 9 |
| Madagascar | MAD066 | 2016 | Maevatanana | N/A | N/A | 13 | N/A | N/A |
| Madagascar | MAD067 | 2016 | Maevatanana | N/A | N/A | 14 | N/A | N/A |
| Madagascar | MAD068 | 2016 | Maevatanana | N/A | N/A | 17 | N/A | N/A |
| Madagascar | MAD069 | 2016 | Maevatanana | N/A | N/A | 18 | N/A | N/A |
| Madagascar | MAD070 | 2016 | Maevatanana | N/A | N/A | 20 | N/A | N/A |
| Madagascar | MAD071 | 2016 | Maevatanana | N/A | N/A | 21 | N/A | N/A |
| Madagascar | MAD072 | 2016 | Maevatanana | N/A | N/A | 21 | N/A | N/A |
| Madagascar | MAD073 | 2016 | Maevatanana | N/A | N/A | 22 | N/A | N/A |
| Madagascar | MAD074 | 2016 | Maevatanana | N/A | N/A | 22 | N/A | N/A |
| Madagascar | MAD075 | 2016 | Maevatanana | N/A | N/A | 22 | N/A | N/A |
| Madagascar | MAD076 | 2016 | Maevatanana | N/A | N/A | 22 | N/A | N/A |
| Madagascar | MAD077 | 2016 | Maevatanana | N/A | N/A | 22 | N/A | N/A |
| Madagascar | MAD078 | 2016 | Maevatanana | N/A | N/A | 22 | N/A | N/A |
| Madagascar | MAD079 | 2016 | Maevatanana | N/A | N/A | 22 | N/A | N/A |
| Madagascar | MAD080 | 2016 | Maevatanana | N/A | N/A | 23 | N/A | N/A |
| Madagascar | MAD081 | 2016 | Maevatanana | N/A | N/A | 24 | N/A | N/A |
| Madagascar | MAD082 | 2016 | Maevatanana | N/A | N/A | 25 | N/A | N/A |
| Madagascar | MAD083 | 2016 | Maevatanana | N/A | 1.19 | 19 | N/A | N/A |
| Madagascar | MAD084 | 2016 | Maevatanana | TCT/CCT | N/A | N/A | 1.43 | N/A |
| Madagascar | MAD085 | 2016 | Maevatanana | TCT/CCT | 0.75 | 14 | 1.45 | 4 |
| Madagascar | MAD086 | 2016 | Maevatanana | TCT/CCT | 1.22 | 14 | 1.44 | 5 |
| Madagascar | MAD087 | 2016 | Maevatanana | TCT/CCT | 1.24 | 10 | 1.31 | C127 |
| Madagascar | MAD088 | 2016 | Maevatanana | TCT | 0.91 | 16 | 1.22 | C127 |
| Madagascar | MAD089 | 2016 | Maevatanana | TCT/CCT | 0.79 | N/A | 1.18 | C127 |
| Madagascar | MAD090 | 2016 | Maevatanana | TCT/CCT | 1.29 | 10 | 0.83 | 9 |
| Madagascar | MAD091 | 2016 | Maevatanana | TCT/CCT | 0.80 | 13 | 1.25 | 9 |
| Madagascar | MAD092 | 2016 | Maevatanana | TCT | 1.06 | 13 | 1.42 | 9 |
| Madagascar | MAD093 | 2016 | Maevatanana | TCT | 0.63 | 13 | 0.94 | N/A |
| Madagascar | MAD094 | 2016 | Maevatanana | TCT/CCT | 0.89 | 16 | 1.43 | N/A |
| Madagascar | MAD095 | 2016 | Maevatanana | TCT/CCT | 0.61 | 17 | 1.69 | 5 |
| Madagascar | MAD096 | 2016 | Maevatanana | TCT/CCT | 0.72 | 14 | 3.07 | 6 |
| Madagascar | MAD097 | 2016 | Maevatanana | TCT/CCT | 0.70 | 10 | 1.57 | C127 |
| Madagascar | MAD098 | 2016 | Maevatanana | TCT/CCT | 1.04 | 16 | 1.83 | C127 |
| Madagascar | MAD099 | 2016 | Maevatanana | TCT/CCT | 0.80 | N/A | 1.60 | C127 |
| Madagascar | MAD100 | 2016 | Maevatanana | TCT/CCT | 0.88 | 10 | 1.55 | 9 |
| Madagascar | MAD101 | 2016 | Maevatanana | TCT/CCT | 1.15 | 13 | 1.70 | N/A |
| Madagascar | MAD102 | 2016 | Maevatanana | N/A | N/A | 13 | N/A | 9 |
| Madagascar | MAD103 | 2016 | Maevatanana | N/A | N/A | 14 | 2.12 | N/A |
| Madagascar | MAD104 | 2016 | Maevatanana | TCT/CCT | N/A | N/A | 1.81 | N/A |
| Madagascar | MAD105 | 2016 | Maevatanana | N/A | N/A | N/A | 2.12 | N/A |
| Madagascar | MAD106 | 2016 | Maevatanana | TCT/CCT | 1.60 | 10 | 0.58 | C127 |
| Madagascar | MAD107 | 2016 | Maevatanana | TCT | 2.31 | 14 | 1.12 | C127 |
| Madagascar | MAD108 | 2016 | Maevatanana | TCT/CCT | 1.53 | 14 | 1.45 | 9 |
| Madagascar | MAD109 | 2016 | Maevatanana | TCT | 2.50 | N/A | 0.51 | N/A |
| Madagascar | MAD110 | 2016 | Maevatanana | TCT/CCT | 1.99 | 14 | 1.63 | N/A |
| Madagascar | MAD111 | 2016 | Maevatanana | N/A | 2.19 | N/A | 1.94 | N/A |
| Madagascar | MAD112 | 2017 | Maevatanana | N/A | N/A | 16 | N/A | N/A |
| Madagascar | MAD113 | 2017 | Maevatanana | TCT/CCT | 1.14 | 13 | 1.06 | C127 |
| Madagascar | MAD114 | 2017 | Maevatanana | TCT/CCT | 1.29 | 14 | 1.20 | 9 |
| Madagascar | MAD115 | 2017 | Maevatanana | TCT/CCT | 1.23 | N/A | 0.90 | N/A |
| Madagascar | MAD116 | 2017 | Maevatanana | N/A | 1.47 | N/A | 1.21 | N/A |
| Madagascar | MAD117 | 2017 | Maevatanana | N/A | 1.26 | 10 | 4.53 | N/A |
| Madagascar | MAD118 | 2017 | Maevatanana | N/A | 1.00 | 13 | 2.69 | N/A |
| Madagascar | MAD119 | 2017 | Maevatanana | N/A | 1.27 | N/A | 2.09 | N/A |
| Madagascar | MAD120 | 2017 | Maevatanana | N/A | 1.29 | N/A | 2.36 | N/A |
| Madagascar | MAD121 | 2017 | Maevatanana | N/A | N/A | 10 | 4.04 | N/A |
| Madagascar | MAD122 | 2017 | Maevatanana | N/A | 4.72 | 14 | N/A | N/A |
| Madagascar | MAD123 | 2017 | Maevatanana | TCT/CCT | 3.57 | 10 | 0.66 | 4 |
| Madagascar | MAD124 | 2017 | Maevatanana | N/A | 3.48 | N/A | 1.36 | N/A |
| Madagascar | MAD125 | 2017 | Maevatanana | N/A | 1.54 | 13 | 1.63 | N/A |
| Madagascar | MAD126 | 2017 | Maevatanana | N/A | 1.93 | 14 | 2.65 | N/A |
| Madagascar | MAD127 | 2017 | Maevatanana | N/A | 1.64 | N/A | 1.72 | N/A |
| Madagascar | MAD128 | 2017 | Maevatanana | N/A | 2.19 | N/A | 1.94 | N/A |
| Madagascar | MAD129 | 2017 | Maevatanana | N/A | 2.73 | N/A | 2.30 | N/A |

N/A: Not available
