# Supplementary material for: Genetic diversity in two Plasmodium vivax protein ligands for reticulocyte invasion
Source: PLoS Negl Trop Dis. 2018 Oct 22;12(10):e0006555. doi: 10.1371/journal.pntd.0006555 (PMC6211765; doi:10.1371/journal.pntd.0006555)
Supplement: S1 Fig — (DOCX) [file pntd.0006555.s005.docx]

***MKATIFCFFFLYLFWSICVASKDVAALSRGENGQAEGSSDISKKKLGVNSKGEGKISSFFQQNGELLEIRKRAVDAIGGTPEKKQSHGEENAEIREDQSFIEMEKANTNHKNGLDESVEEALNEGESFIEDKSDRDSAPLVEDPTKGDENRDHAEGADT***LTSSVNNGKIPTNNVAKYVFSDTSLISGCNAKREREKWYC**N**LNGVQEQDICIPDRRAQM**C**INNLVNVKSGNEK**N**DLKEQVLLSLNTESQLLFNKWKKHNSFNNEEFCN**D**LNRDYADFGNLIKGTDIVAHGNSKEVEDKLKQIFGEN**E**NAKS**D**REKWWNDNKE**E**FWNKLLSSVKGKGKEGNVEIKECTKDATLEEIPQFQRWVQEWGKEYGEERPKKLQNLEGICKEKNGLLNENRCNNEHECKRTCTAYESWIILKKEQWD**T**I**S**KKYDIVKDDEKYVKKGSNETAMKFL**N**ELCKECNLGDFEKI**I**NLKDDEYTKLCNCQVHKGVKIAGGSTKDENRGDPTSHEIRSHEGGSRAAVNGQRDDAGRVREAP***EVDENGKSKGRGALDRNSINPTNEGDSVRTGKNKVTRGREGIDSTEVEDAKSDVAQKEGSSVPSQIKGSETRDARGKSSKEQISASKQGNAEEERDLQVHNFSRTNALNGRSNSVRSQNVQSRGKEKSDREEGGNKDQHKDDGYSNAQYNNLNVQDSAGRNGSRRTEALHSLWKNSVCNNYNTPESCENAKGLNSSEDNSNDDEKRVCCSIVGYCLKFFDHNSREYNYCVEKEYYDSLQNSSKQGFPTGILYFAAGGLFIVLAAFAVAANKSGNM***
